# Ten simple rules to make computable knowledge shareable and reusable

**DOI:** 10.1371/journal.pcbi.1012179

**Published:** 2024-06-20

**Authors:** Marisa L. Conte, Peter Boisvert, Philip Barrison, Farid Seifi, Zach Landis-Lewis, Allen Flynn, Charles P. Friedman

**Affiliations:** Department of Learning Health Sciences, University of Michigan Medical School, Ann Arbor, Michigan, United States of America; Dassault Systemes BIOVIA, UNITED STATES

## Abstract

Computable biomedical knowledge (CBK) is: “the result of an analytic and/or deliberative process about human health, or affecting human health, that is explicit, and therefore can be represented and reasned upon using logic, formal standards, and mathematical approaches.” Representing biomedical knowledge in a machine-interpretable, computable form increases its ability to be discovered, accessed, understood, and deployed. Computable knowledge artifacts can greatly advance the potential for implementation, reproducibility, or extension of the knowledge by users, who may include practitioners, researchers, and learners. Enriching computable knowledge artifacts may help facilitate reuse and translation into practice. Following the examples of 10 Simple Rules papers for scientific code, software, and applications, we present 10 Simple Rules intended to make shared computable knowledge artifacts more useful and reusable. These rules are mainly for researchers and their teams who have decided that sharing their computable knowledge is important, who wish to go beyond simply describing results, algorithms, or models via traditional publication pathways, and who want to both make their research findings more accessible, and to help others use their computable knowledge. These rules are roughly organized into 3 categories: planning, engineering, and documentation. Finally, while many of the following examples are of computable knowledge in biomedical domains, these rules are generalizable to computable knowledge in any research domain.

## Author summary

Computable biomedical knowledge, including algorithms, models, and evidence, is already impacting—and will likely transform—health research and healthcare delivery. Representing this knowledge in formats that can be discovered and processed by computers increases its potential for use at scale. Similarly, making these algorithms, models, etc. (aka computable knowledge artifacts) understandable and reusable by others can help facilitate reuse and translation into practice. However, as with other types of scientific software or research data, creating artifacts that are usable by and useful to others requires intentionality, and often additional time and effort. We’ve developed over 100 computable knowledge artifacts in diverse areas of health research and healthcare and we’ve learned a few things along the way. We’ve distilled these lessons learned into 10 Simple Rules to help researchers who would like to make their computational research products available and useful to others. Our work centers on knowledge in the biomedical domain, but we believe these 10 Simple Rules are generalizable and valuable as design principles for any researcher wishing to share computational research products.

## Introduction

Computable biomedical knowledge (CBK) is variously defined as: “the [explicit] result of an analytic and/or deliberative process about human health … that can be represented and reasoned upon using logic, formal standards, and mathematical approaches”[1], and “software artifacts containing machine-interpretable or executable instructions that transform input data into practical outputs”[2]. We note the essential difference between “computer-*based* knowledge, which is held and shared in text format for computers to read … and computer-*executable* (computable) knowledge, which is held in a format that can be reasoned with or applied by the computer to carry out a task”[3] (italics in original), and use the term “computable knowledge” for executable CBK artifacts. CBK may have diverse representations, e.g., mathematical functions, or machine learning models, and implementations, e.g., algorithms, practice guidelines, predictive or classification models, and may be shared in many ways, from a GitHub repository to a deployable container. Representing biomedical knowledge in computable forms increases discoverability and use—even a rudimentary CBK artifact can advance the potential for implementation, reproducibility, or extension of the knowledge it contains.

Properly developed, validated, implemented, and stewarded, CBK can:

- Accelerate knowledge translation: CBK has the potential to reduce the commonly cited 17-year gap [4] between knowledge discovery and its application, keep pace with new knowledge generation, and facilitate the translation of knowledge into practice at scale.
- Improve healthcare delivery: Waste and inefficiency in US healthcare burdens patients, providers, health systems, and communities [5]. Harnessing CBK may facilitate improvement in care delivery, resource utilization, and research [6].
- Enable the learning health system: CBK has been recognized as an imperative for learning health systems [7,8], which are characterized by their ability to generate, collect, and learn from internal data to improve practice.

With this growing potential for CBK comes the increased importance for sharing CBK artifacts to facilitate knowledge understanding and use at scale. We have developed an ontology-specified [9] knowledge object (KO) model that packages CBK with metadata and implementation information [10], and created over 100 KOs [11]. Along the way, we’ve learned how to enrich computable knowledge artifacts to facilitate sharing and reuse.

We’ve distilled this into 10 simple rules for making computable knowledge shareable and reusable. While the examples below focus on computable knowledge in clinical research and practice, these rules are generalizable to computable knowledge in any research domain. Sharing computable knowledge is related to other efforts to increase the transparency, reproducibility, and reusability of research, including data sharing and reproducible workflows [12] and semantic publications.

## Ten simple rules

**Fig 1:**
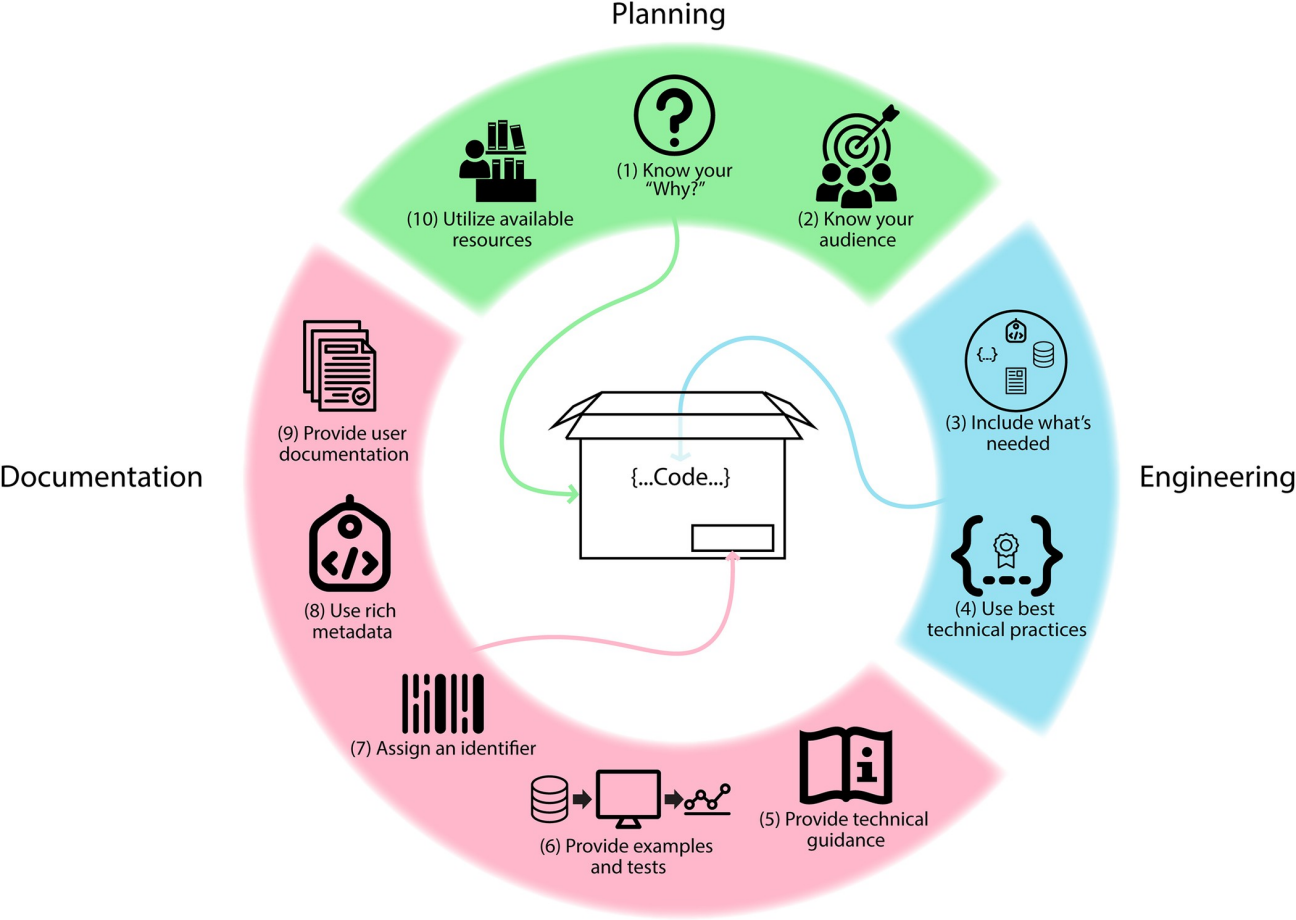
Ten Simple Rules for making computable knowledge shareable and reusable. The Rules fall in 3 categories: planning, engineering, and documentation. The source for all of the clipart is the NounProject (https://thenounproject.com/).

Following the examples of 10 Simple Rules papers for scientific software [13,14] and applications [15], writing about scientific software [16], and working with other people’s code [17], our rules are intended to make computable knowledge artifacts more shareable, useful, and reusable. These rules are useful for researchers who wish to go beyond traditional publication pathways for knowledge dissemination, and who want to both make their artifacts more accessible, and to help others use them. These rules are organized into 3 categories (Fig 1).

## Rule 1: Determine if your computable knowledge is appropriate for sharing—Know your “why?”

Sharing computable knowledge requires intentionality, and it can be helpful to know your motivations for sharing. Is it to make a research process transparent and reproducible, standardize an approach, promote implementation, facilitate additional scientific discovery, contribute to building a community of practice? There are many use cases for sharing knowledge, including research reproducibility or replication, use in teaching, or further study of the knowledge or artifacts themselves. Sharing also requires time and additional work to ensure that your knowledge is accessible, understandable—and ideally useful—to others. We encourage researchers to consider whether this is a reasonable course of action. Some questions that may guide this decision are shown in Table 1.

**Table 1:** Factors that may influence your decision to share a knowledge artifact.

• Are you required to share research products as a condition of your funding? • Do you see potential for reuse of this artifact, by yourself or others? • Is there potential to integrate this artifact into other systems, products, or applications? • Is your artifact at a stable state or do you anticipate making further revisions in the short term? • Does your artifact work with open resources or only with proprietary resources? • Is your knowledge artifact highly localized or do you see potential for generalizability? • Will sharing this knowledge artifact make it easier for you to collaborate with or train others? • Does sharing this artifact advance your research or your professional goals (e.g., could it help to attract collaborators or possible industry support, or demonstrate your skills and proficiency in a way that establishes you in a community)? • Does sharing this artifact demonstrate your professional values, e.g., open science, transparency, rigor, and reproducibility?

Another decision is with whom your knowledge artifact will be shared. This may be at your discretion, or other factors, e.g., funder or institutional policies, may guide your decision-making. It’s worth noting here that “shared” and “open” are not necessarily equivalent—sharing can take many forms, including permitting only certain types of reuse, or specific users, i.e., collaborators within a research network, or a proscribed community of practice. Select a software license for your computable knowledge that is compatible with your intended users and use cases, and consider permissive open source licenses where possible (for assistance with licensing see, e.g., Rule 10 and resources like the Turing Way [18]). Together with other 10 Simple Rules authors who have written about the benefits of open source software [13,14,19], we believe that open is good. We also acknowledge it’s sometimes impractical or impossible.

## Rule 2: Know your audience

Most computable knowledge is designed either for a specific purpose (e.g., clinical decision support) or for a specific community (e.g., researchers who develop computational models). Clearly articulating both the intended user community and use context helps in several ways.

A solid understanding of user needs and requirements can inform decisions about how you engineer your knowledge artifact and can help identify other elements that may increase the potential for discovery and reuse. Additionally, describing the intended users/use may help others determine the suitability of the artifact for their purposes.

As a starting point, consider how this artifact can propel future work, or address current or anticipated needs of your collaborators, or community, then consider how others outside of your immediate circle might use this artifact. Taking a more expansive look at potential contexts and uses will lead you to make different decisions about things that facilitate understanding or implementation than if you consider the potential of your knowledge artifact through a narrower lens.

For example, our lab developed a collection of KOs based on the Clinical Pharmacogenomics Implementation Consortium’s (CPIC) guidelines for integrating genetic tests and prescribing (https://kgrid-objects.github.io/cpic-collection/). There are both clinical and research use cases, and clinicians and researchers may require different implementations of the core knowledge (e.g., individual patient versus batch processing) and may benefit from different types of metadata (e.g., a clinician may appreciate references to current evidence in the documentation).

## Rule 3: Design your computable knowledge artifact to include everything that is needed for computation

All components required for computing should be directly available in your artifact, either as part of the artifact or via persistent links to other materials. Anticipate future use and include what would be needed to understand and interpret the knowledge in the most common use cases. Then, consider what information is typically used in conjunction with this knowledge, or is needed to interpret outputs, etc., and decide which of these should be incorporated into your computable knowledge artifact. For example, we created a KO to score, interpret, and provide recommendations based on the Patient Health Questionnaire 9 (PHQ-9), a validated instrument to diagnose, assess, and measure the severity of depression [20]. Three components must be included for this artifact to be useable: survey questions, value sets of responses, and recommendations corresponding to each possible score. Using standard representations can help with interoperability and reusability; examples from the clinical domain include Clinical Quality Language (CQL) and Fast Healthcare Interoperability Resources (FHIR).

## Rule 4: Design your computable knowledge artifact using best technical practices for your target integrations

Following best technical practices for code organization, design, and packaging can significantly affect the potential for reuse of your knowledge artifact by others, making it easier to understand and apply, and potentially more interoperable with existing systems. Platforms like GitHub offer best practice recommendations or templates, and resources, e.g., Cookiecutter Data Science (https://drivendata.github.io/cookiecutter-data-science/) and the Turing Way [18] provide guidance for structuring a project. In many cases, automated processes may consume and implement computable knowledge, provide machine-interpretable artifacts (e.g., Dockerfiles, JSON-LD metadata, OpenAPI definitions) to help with automated deployment, indexing, tooling, etc.

## Rule 5: Provide technical guidance for implementation

This Simple Rule is for the implementers—those whose task is to get the computable knowledge working in a system other than the one for which it was developed. One purpose of computable knowledge is to add capabilities to modern information technology systems, and this will usually require some degree of human intervention. This human intervener might be a downstream developer, a health IT implementer, or a researcher replicating and extending a previous result. While your code may work well for you in your own environment, this may not necessarily be the experience of a new user [17]. In some cases, packaging your computable knowledge (e.g., for a platform, like a Jupyter notebook or providing a Docker container) can make it easier to implement. In other cases, ensure that it’s easy for users to find the right platform or environment in which to deploy your knowledge artifact. Additionally, provide robust documentation—this may include a run script, container image, download links for tools, implementation guide, or step-by-step instructions. As there is usually more than one “right way” to make things work, it may be helpful to provide and document alternatives, while adhering to best practices for the chosen tech stack.

## Rule 6: Provide examples and tests

Provide examples, prototypes, or sample client software; a full client can be ready to run or can serve as a base for custom integrations. Showing your computable knowledge artifact in action can help others understand how it functions; examples can also highlight good practices for using your computable knowledge.

Having more than 1 example or client will help potential users or implementers. It will also deepen your understanding of your core knowledge and provide opportunities for improvement. For example, developing a second client may expose model limitations that would not have been otherwise identified.

Providing robust and comprehensive tests demonstrates that the code powering your computable knowledge artifact does what it claims to do [17]. Unit and integration tests typically used in software development are a great start, but users may also appreciate a validation suite. Providing sample input data with corresponding output results can help users test the artifact. Tests are the first step towards reproducibility and replicability, a key element of establishing trust, and can help people understand your computable knowledge. They are also extremely useful for editors or peer reviewers in cases where you intend to publish your artifact (e.g., as a software paper).

## Rule 7: Assign a unique identifier to make your computable knowledge findable and accessible

Making computable knowledge shareable requires time and effort, so maximizing the potential for use/reuse is important. One of the most important things you can do to make your computable knowledge findable and accessible is to assign a persistent identifier. Persistent identifiers reference your computable knowledge and make it machine-retrievable. Examples of common persistent identifiers include the Archival Resource Key (ARK: https://arks.org/), Digital Object Identifier (DOI: https://www.doi.org/), and Handle (http://www.handle.net/). Persistent identifiers can be assigned through institutional affiliation with registration services, or via repositories or platforms such as Zenodo (https://zenodo.org/).

These identifiers can be included with the metadata for your computable knowledge, and referenced at multiple places to make your work findable and accessible, including lab websites, GitHub repositories and in relevant publications. Also consider providing a formatted citation. This can be done as text, but some platforms also provide for machine-readable citation files; GitHub offers a CITATION.cff file (https://citation-file-format.github.io/) that represents useful information and metadata, and that can be stored and used in citation managers, linked in repositories, and ingested by registries.

## Rule 8: Use rich metadata to help people (and machines) discover, understand, and manage your computable knowledge artifact

Robust metadata can also increase the findability and potential for the reuse of your computable knowledge artifact. Metadata is commonly called “data about data” [21], but for our purposes can be better understood as machine-readable documentation of the administrative, descriptive, and technical properties of your computable knowledge. Metadata is the way to make your computable knowledge artifact findable, accessible, and usable by machines, and by the people who use machines to find, access, and use computable knowledge. Machine-interpretable metadata can be used by search engines and indexing services and can provide a standardized way to query domain-specific terms across resources or repositories.

It’s easy to be overwhelmed by metadata, so we recommend that you start with the basics (and know that there is help available—see Rule 10). First, describe the *who*, *what*, *when*, and *where* of your computable knowledge: Who created the artifact? What version is this? When was it created? Where did this knowledge come from (e.g., is this knowledge derived from another artifact? Are there existing evidence sources to point to?)? Then, describe the why, or the purpose for which your knowledge was created, and the context in which it’s intended to be used. Communicating the purpose of the knowledge representation is important for reuse as it helps users determine the relevance or applicability to their own needs or use cases.

In addition to metadata describing the purpose, you can also include descriptive metadata relevant to the specific domain of your computable knowledge. Here, using standardized terminologies can be helpful for machine indexing and retrieval. For example, if you’ve developed a cancer risk prediction model, consider preferred terms from the National Cancer Institute Thesaurus (https://ncithesaurus.nci.nih.gov/ncitbrowser/) to describe clinical features, diagnoses, or other factors relevant to your model. Communities of practice may also have required or suggested metadata; for example, authors of CBK artifacts may wish to consider the metadata categories proposed by the MCBK community [22].

Finally, linked data conventions [23] are an opportunity to add context and allow you to link to valuable external resources. To derive the most benefit from linked data, explore existing ontologies and terminologies that can be used to standardize your terms.

## Rule 9: Provide robust user documentation

In addition to machine-readable metadata, human-readable documentation is also important. Comments in code, a readme, or a documentation website with FAQs are all ways to help users of varying abilities understand your computable knowledge, and documentation is a likely place for a new user to look while attempting to understand the code behind your knowledge [17]. This documentation is related to Rule 5’s technical guidance for implementation, but covers more ground than just getting the artifact running, and is intended primarily for the end-user who will need to understand, among other things, how the knowledge functions.

While this may sound like a lot of work to do for someone else, this documentation can help you, too, or someone in your lab. Picture yourself a year or so in the future, when you find that all the tacit knowledge you had at the front of your mind while working with this code daily has dissipated. You will appreciate having thorough documentation when you return to reproduce, replicate, or extend your past work.

## Rule 10: Utilize available expertise and resources

While the above rules can help make your computable knowledge more accessible and available for reuse, sharing these artifacts will require you to expend time and effort. Understand what resources are available to you, e.g., through funders or your institution, to assist in the work of making your computable knowledge ready to share and possible to reuse.

Academic librarians are an excellent resource for questions about persistent identifiers, metadata standards, domain terminologies, and other issues related to making your computable knowledge findable by search engines or indexing services. Many academic libraries now provide data management services, and there is significant potential to extend existing tools and best practices used in curating datasets to computable knowledge. A librarian with domain expertise may also be able to suggest a reputable digital repository for your knowledge artifact. This may be an institutional repository such as the University of Michigan’s Deep Blue (https://www.lib.umich.edu/collections/deep-blue-repositories), a generalist repository like Zenodo (www.zenodo.org), or a domain-specific repository such as EMBL-EBI’s BioModels (https://www.ebi.ac.uk/biomodels/).

Your institution or funder may also offer resources or services that can provide guidance for issues pertaining to your computable knowledge. For example, if you have concerns about licensing, intellectual property, liability, or restricting access, consider consulting with your institution’s technology licensing or technology transfer office to ensure your plans to share your knowledge seem reasonable and in line with other considerations (e.g., future directions of your work).

## Conclusions

There is great potential benefit from preparing computable knowledge for sharing and reuse. However, these benefits come at a cost—the necessary or recommended activities require time and effort, and there are currently few incentives for these investments. As peer-reviewed papers are currently highly valued scientific contributions, academic researchers are more incentivized to publish research papers in peer-reviewed journals than to share code, workflows, or other research outputs. Additionally, making computable knowledge shareable involves skillsets that aren’t currently present in many research labs, including expertise in metadata schemas, ontologies, and data standards. Finally, the infrastructure to create an ecosystem for stewardship of computable knowledge artifacts is in its infancy—currently, there is no centralized library or registry of computable knowledge, and no easy way to centrally manage these resources through their lifecycles.

Despite these challenges, the benefits of sharing computable knowledge will become increasingly apparent as artificial intelligence, machine learning, and other computational methods become increasingly prominent in biomedical research and clinical care. As more attention is paid to the value of sharing knowledge, incentives will likely shift to reflect this reality. In the meantime, the 10 simple rules outlined here can act as a starting point for researchers and developers of computable knowledge to make their artifacts more shareable and reusable.

## References

[1] Mobilizing Computable Biomedical Knowledge (MCBK). MANIFESTO. [cited 2021 Jan 22]. Available from: https://mobilizecbk.med.umich.edu/about/manifesto.

[2] Koru G. Transforming health and well-being through publishing computable biomedical knowledge (CBK). Learn Health Syst. 2023;7:e10396. doi: 10.1002/lrh2.10396 37860055 PMC10582207

[3] Wyatt J, Scott P. Computable knowledge is the enemy of disease. BMJ Health Care Inform. 2020;27:e100200. doi: 10.1136/bmjhci-2020-100200 32723856 PMC7388882

[4] Morris ZS, Wooding S, Grant J. The answer is 17 years, what is the question: understanding time lags in translational research. J R Soc Med. 2011;104:510–520. doi: 10.1258/jrsm.2011.110180 22179294 PMC3241518

[5] Delaune J, Everett W. Waste and inefficiency in the US health care system: Clinical care: a comprehensive analysis in support of system-wide improvements. New England Healthcare Institute. 2008. Available from: https://www.nehi-us.org/writable/publication_files/file/waste_clinical_care_report_final.pdf.

[6] Shrank WH, Rogstad TL, Parekh N. Waste in the US Health Care System: Estimated Costs and Potential for Savings. JAMA. 2019;322:1501. doi: 10.1001/jama.2019.13978 31589283

[7] Lehmann HP, Downs SM. Desiderata for sharable computable biomedical knowledge for learning health systems. Learn Health Syst. 2018;2:e10065. doi: 10.1002/lrh2.10065 31245589 PMC6508769

[8] Friedman CP, Flynn AJ. Computable knowledge: An imperative for Learning Health Systems. Learn Health Syst. 2019:3. doi: 10.1002/lrh2.10203 31641690 PMC6802532

[9] Flynn AJ, Friedman CP, Boisvert P, Landis-Lewis Z, Lagoze C. The Knowledge Object Reference Ontology (KORO): A formalism to support management and sharing of computable biomedical knowledge for learning health systems. Learn Health Syst. 2018;2:e10054. doi: 10.1002/lrh2.10054 31245583 PMC6508779

[10] Flynn A, Taksler G, Caverly T, Beck A, Boisvert P, Boonstra P, et al. CBK model composition using paired web services and executable functions: A demonstration for individualizing preventive services. Learn Health Syst. 2023;7:e10325. doi: 10.1002/lrh2.10325 37066102 PMC10091204

[11] Flynn A, Conte M, Boisvert P, Richesson R, Landis-Lewis Z, Friedman C. Linked Metadata for FAIR Digital Objects Carrying Computable Knowledge. Res Ideas Outcomes. 2022;8:e94438. doi: 10.3897/rio.8.e94438

[12] Soiland-Reyes S, Sefton P, Crosas M, Castro LJ, Coppens F, Fernández JM, et al. Packaging research artefacts with RO-Crate. Data Sci. 2022;5:97–138. doi: 10.3233/DS-210053

[13] Prlić A, Procter JB. Ten Simple Rules for the Open Development of Scientific Software. PLoS Comput Biol. 2012;8:e1002802. doi: 10.1371/journal.pcbi.1002802 23236269 PMC3516539

[14] Hunter-Zinck H, de Siqueira AF, Vásquez VN, Barnes R, Martinez CC. Ten simple rules on writing clean and reliable open-source scientific software. PLoS Comput Biol. 2021;17:e1009481. doi: 10.1371/journal.pcbi.1009481 34762641 PMC8584773

[15] Burnett JL, Dale R, Hou C-Y, Palomo-Munoz G, Whitney KS, Aulenbach S, et al. Ten simple rules for creating a scientific web application. PLoS Comput Biol. 2021;17:e1009574. doi: 10.1371/journal.pcbi.1009574 34882674 PMC8659688

[16] Romano JD, Moore JH. Ten simple rules for writing a paper about scientific software. PLoS Comput Biol. 2020;16:e1008390. doi: 10.1371/journal.pcbi.1008390 33180774 PMC7660560

[17] Pilgrim C, Kent P, Hosseini K, Chalstrey E. Ten simple rules for working with other people’s code. PLoS Comput Biol. 2023;19:e1011031. doi: 10.1371/journal.pcbi.1011031 37079492 PMC10118162

[18] Community TTW. The Turing Way: A handbook for reproducible, ethical and collaborative research. Zenodo. 2022. doi: 10.5281/zenodo.7625728

[19] Boland MR, Karczewski KJ, Tatonetti NP. Ten Simple Rules to Enable Multi-site Collaborations through Data Sharing. PLoS Comput Biol. 2017;13:e1005278. doi: 10.1371/journal.pcbi.1005278 28103227 PMC5245793

[20] Kroenke K, Spitzer RL, Williams JBW. The PHQ-9. J Gen Intern Med. 2001;16:606–613. doi: 10.1046/j.1525-1497.2001.016009606.x 11556941 PMC1495268

[21] Baca M. Introduction to Metadata. 20 Jul 2016 [cited 2020 Apr 27]. Available from: http://www.getty.edu/publications/intrometadata.

[22] Alper BS, Flynn A, Bray BE, Conte ML, Eldredge C, Gold S, et al. Categorizing metadata to help mobilize computable biomedical knowledge. Learn Health Syst. 2022;6:e10271. doi: 10.1002/lrh2.10271 35036552 PMC8753304

[23] Bizer C, Heath T, Idehen K, Berners-Lee T. Linked data on the web (LDOW2008). Proceedings of the 17th international conference on World Wide Web. New York, NY, USA: Association for Computing Machinery; 2008. p. 1265–1266. doi: 10.1145/1367497.1367760

